# Evaluation of effectiveness and improvement factors of occupational health and safety management system in the Republic of Korea Navy based on AHP-entropy and IPA

**DOI:** 10.1371/journal.pone.0283653

**Published:** 2023-04-04

**Authors:** Sang ji Lee, Yun-Hee Choi, Da-An Huh, Seok Joon Yoon, Kyong Whan Moon

**Affiliations:** 1 Republic of Korea Navy Headquarters, Republic of Korea; 2 Department of Health and Safety Convergence Science, Korea University, Seoul, Republic of Korea; 3 BK21 FOUR R&E Center for Learning Health System, Korea University, Seoul, Republic of Korea; 4 Institute of Health Sciences, Korea University, Seoul, Republic of Korea; 5 Department of Health and Environmental Science, Korea University, Seoul, Republic of Korea; University of Siena: Universita degli Studi di Siena, ITALY

## Abstract

The Republic of Korea Navy (ROKN) has applied Occupational Health and Safety Management System (OHSMS), but the effectiveness of OHSMS is being questioned due to continuous industrial accidents that continue to occur. Although OHSMS, which has been generally applied in enterprises, has more potential for improper implementation in the military, there are few studies on OHSMS in the military. Therefore, this study verified the effectiveness of OHSMS in the ROKN and derived improvement factors. This study was conducted in a two-step process. First, we surveyed 629 workers at the ROKN workplaces to confirm the effectiveness of OHSMS by comparing occupational health and safety (OHS) efforts according to whether OHSMS was applied and the period of application. Second, 29 naval OHSMS experts evaluated the factors for improving OHSMS using two decision-making tools: Analytic Hierarchy Process (AHP)-entropy and Importance-Performance Analysis (IPA). The study results indicate that the OHS efforts of OHSMS-applied workplaces were similar to that of unapplied. Also, no better OHS efforts were identified in workplaces with more extended OHSMS application periods. There were five improvement factors of OHSMS applied to the ROKN workplaces, with the highest weight in the following order: consultation and participation of workers; resources; competence; hazard identification and risk assessment; and organizational roles, responsibilities, and authorities. The effectiveness of OHSMS in the ROKN was insufficient. Therefore, the ROKN needs focused improvement efforts on the five requirements to implement OHSMS practically. These results can be helpful information for the ROKN to apply OHSMS more effectively for industrial safety.

## Introduction

As a work environment free from work-related damage or disease has become essential, the system to protect the health and safety of workers has been introduced. Occupational Health and Safety Management System (OHSMS) was developed to predict and prevent health and safety issues that may occur in organizations through the plan-do-check-act activities and ultimately incorporate their risk management systematically [1]. In addition, OHSMS has served as a certification tool that demonstrates the transparency and competitiveness of a company by documenting the company’s physical and human resources and their organizations, responsibilities, and procedures managing them [2]. Since 2018, as OHSMS was formally enacted as ISO-45001, an international standard, it is emerging as an essential management strategy for companies [3], and more and more organizations are applying OHSMS [4].

This popularity of OHSMS also extends to the militaries, which have conducted occupational health and safety (OHS) management in a state of being closed and separated from society. In the military, more than 40% of the workforce participates in industrial activities for military support. And daily work accidents in these industries often inflict damage beyond combat losses. For example, in the US military, the life lost due to daily work (31.9%) exceeded the combat loss (16.3%) in 2006–2018 [5]. Also, in the Republic of Korea (ROK) military, the occupational accident rate (0.4%) was like that of Korean general workplaces (0.5%) in 2019–2020 [6]. For this reason, global militaries have systematized OHS management by modifying the systems according to ISO-45001 and verifying them by external agencies [7–9]. Among the ROK military, the ROK Navy (ROKN) recognized the need to improve OHS management relatively early due to dangerous work environments (e.g., ship support and pier work) and first introduced OHSMS in 2015 [10]. The ROKN has promoted the OHSMS certification business in earnest since 2018; as a result, 61.9% of all workplaces have successfully applied OHSMS [10]. However, tangible results are insufficient despite the ROKN’s efforts to improve the OHS. Work accidents more than tripled in 2020 compared to 2019, and human and resource damages have doubled and 8.5 times, respectively, though the number of OHSMS-certified workplaces has steadily increased from 28.6% to 52.4% in 2018–2020 [11]. As a result, questions are being raised as to whether the application of OHSMS a practical effect in has improving the ROKN’s safety level and preventing accidents.

In general, the successful prevention of occupational accidents and implementation of OHS through OHSMS is not achieved by system introduction but must be accompanied by continuous review and improvement [12]. Several studies have revealed that OHSMS-certified workplaces used OHSMS only for social legitimacy and failed to develop a systematic process for OHS management, resulting unsafe environment [13, 14]. Therefore, to derive improvement factors for OHSMS, several analytic hierarchy processes (AHP)-based methods (e.g., fuzzy-AHP, AHP-Technique for Order Preference by Similarity to Ideal Solution (TOPSIS), fuzzy AHP-VlseKriterijuska Optimizacija I Komoromisno Resenje (VOIKR), and AHP-entropy) are being used [15–20]. AHP can quantify OHSMS improvement factors those workers subjectively answered, by developing a hierarchical representation of a decision problem, calculating weights through pairwise comparison of hierarchical elements, and determining their relative importance [18]. However, AHP is still limited in that it only calculates the importance and priority of attributes rather than performance. Therefore, methods capable of assessing both factors (i.e., importance-performance analysis (IPA)) were newly proposed in other decision-making problems [21–24]. Despite IPA’s usefulness, only some studies have applied them in OHSMS [21, 22]. Moreover, as ROKN has had no tangible safety results since the introduction of OHSMS, it is necessary to establish improvement measures through the above methods.

The ROKN already figured out the following problems while introducing OHSMS: difficulty in the examination caused by the lack of understanding of the ROKN’s unique working environment by external assessors and scarcity of internal experts for system operation, etc. [25]. The ROKN officials predicted that due to these problems, tangible results of the standard system did not appear, and its effectiveness would also be insufficient. Nevertheless, empirical confirmation and improvement on these problems have not been made, and as far as we know, no research has been conducted on the ROKN’s application of OHSMS. Therefore, this study tried to verify the effectiveness of OHSMS in the ROKN, identify the need for improvement, and derive improvement factors of OHSMS using scientific models.

## Materials and methods

### Study framework and participants

**Fig 1:**
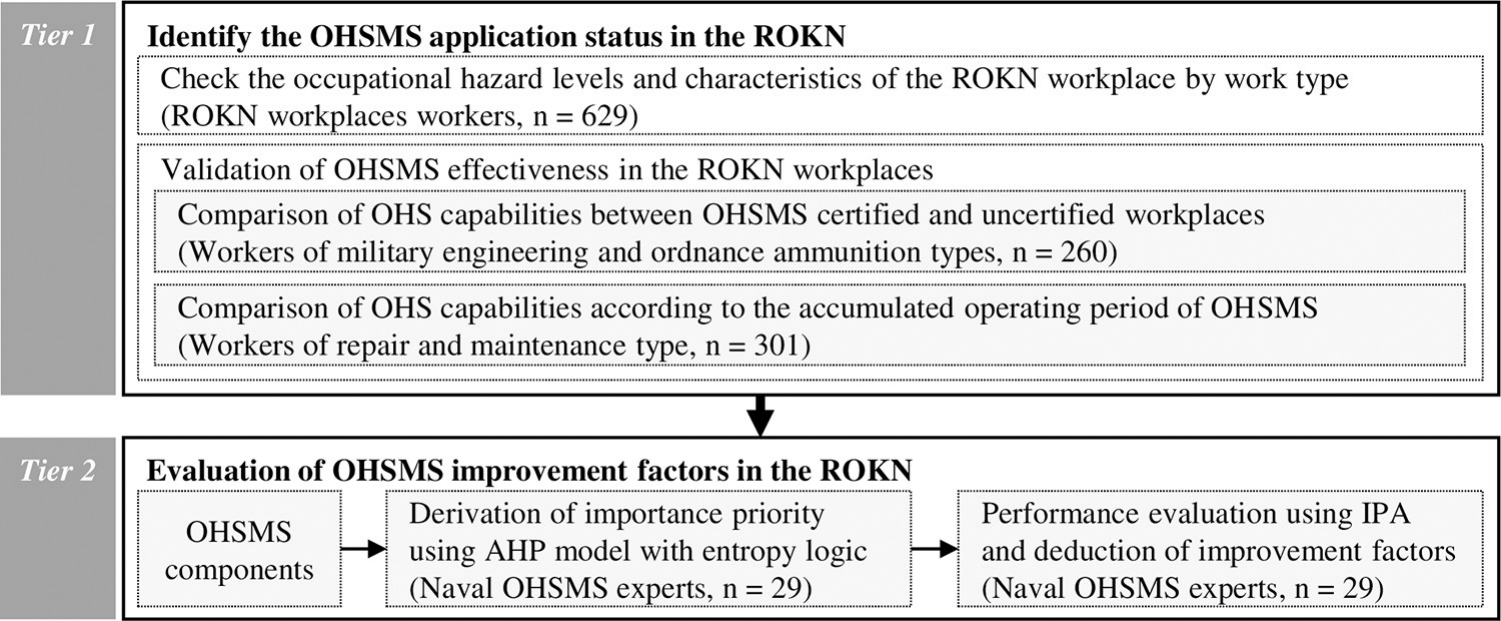
Study framework and participants.

The study was conducted in two processes (Fig 1): tier 1, Confirmation of OHSMS application in the ROKN; and tier 2, Evaluation of improvement factors to improve the effectiveness of OHSMS in the ROKN. Tier 1 started with understanding OHSMS application environment by identifying the occupational hazard levels and characteristics of the ROKN workplaces. Then, the effectiveness of OHSMS, the core of tier 1, was checked according to whether OHSMS was applied and the application period. In tier 2, AHP-entropy and IPA analysis were used to evaluate improvement factors for improving the effectiveness of OHSMS in the ROKN. We collected data based on the survey at all tiers. However, the participants and contents of the survey differed at each tier by purpose.

In tier 1, the occupational hazard factors and application effects of OHSMS were identified by investigating the workers in the ROKN workplaces. We aimed to survey the population representing 10% of the total personnel of the ROKN workplaces. Then, considering the non-responding rate of the survey (15%), 646 people were selected as the study subjects. The study subjects were recruited by systematic sampling within the ROKN workplace after distributing proportions according to the size of each workplace type (repair and maintenance, about 50%; military engineering, about 20%; ordnance ammunition, about 20%; military logistics, about 10%). Among recruited 646 participants, 629 were finally selected for tier 1 study, excluding 17 who missed the survey or responded incorrectly. Then, we compared the demographic and working characteristics of the study participants with those of the entire population to confirm that the selected sample is representative of the ROKN industrial population. As a result, no difference in the characteristics was observed between the two groups, so the selected sample represented the entire ROKN industrial population (S1 Table in S1 File).

In tier 2, the improvement factors in OHSMS application were derived from naval experts who performed OHSMS certification and maintenance tasks. To properly evaluate improvement factors, participants who experienced and had knowledge in the relevant field were needed. Therefore, over one year, those who had experience in acquiring or maintaining OHSMS in the ROKN were selected as study subjects. Among the 50 experts who satisfied the conditions, 29 were selected as the final study participants, excluding 21 who did not agree with the survey.

### Questionnaire survey

The questionnaires were divided into two types: type A (tier 1), a questionnaire to confirm occupational hazard characteristics and OHS efforts of workplaces; and type B (tier 2), a questionnaire to identify improvements in OHSMS. Type A questionnaire items consisted of occupational hazard factors (n = 20) and OHS efforts (n = 18) extracted from the Occupational Safety and Health Company Survey designed by the Korea Occupational Safety and Health Agency (KOSHA) for ROK companies (Table 1). All items were rated on a 5-point Likert scale. Occupational hazard factors were marked with a high score according to the degree to which the evaluator considered it dangerous (1–5 points, ‘very safe’ to ‘very dangerous’), and OHS efforts were marked with a high score according to the degree of excellence that the evaluator considered (1–5 points, ‘very low’ to ‘very high’).

**Table 1:** Occupational hazard factors and OHS efforts questionnaire items (number of questions).

Division	Fields	Items
Occupational hazard factors (n = 20)	Biochemicals (n = 5)	1) Microbial, viral infection
		2) Skin contact with chemical
		3) Hazardous gas, dust, fumes, mist
		4) Explosion, fire material
		5) Heat (flames, hot, liquids, cooling)
	Physical burden (n = 5)	6) Long-standing posture
		7) Prolonged sitting posture
		8) Unnatural posture
		9) Heavy lifting
		10) Repetitive action
	Mechanical/electric (n = 3)	11) Mechanism, equipment
		12) Voltage, electric shock
		13) Vehicle
	Work environment (n = 7)	14) Noise
		15) Extreme temperature
		16) Vibration
		17) Confined space work
		18) Slip, stumble, fall
		19) Collapse
		20) Radiation, harmful rays
OHS efforts (n = 18)	OHS system (n = 6)	1) OHS management
		2) Participation
		3) Roles and responsibility
		4) Qualification and competency
		5) Resource supports
		6) Contribution of OHS management system
	OHS management (n = 7)	7) Communication
		8) Suggestion system
		9) Response to needs
		10) Training
		11) Effect of training
		12) Regulations and procedures
		13) Effect of regulations and procedures
	Accident prevention activities (n = 5)	14) Facility, equipment, and protective equipment
		15) Clean up the hazardous areas
		16) Work under safety procedures and standards
		17) Stop hazardous work
		18) Voluntary participation

**Fig 2:**
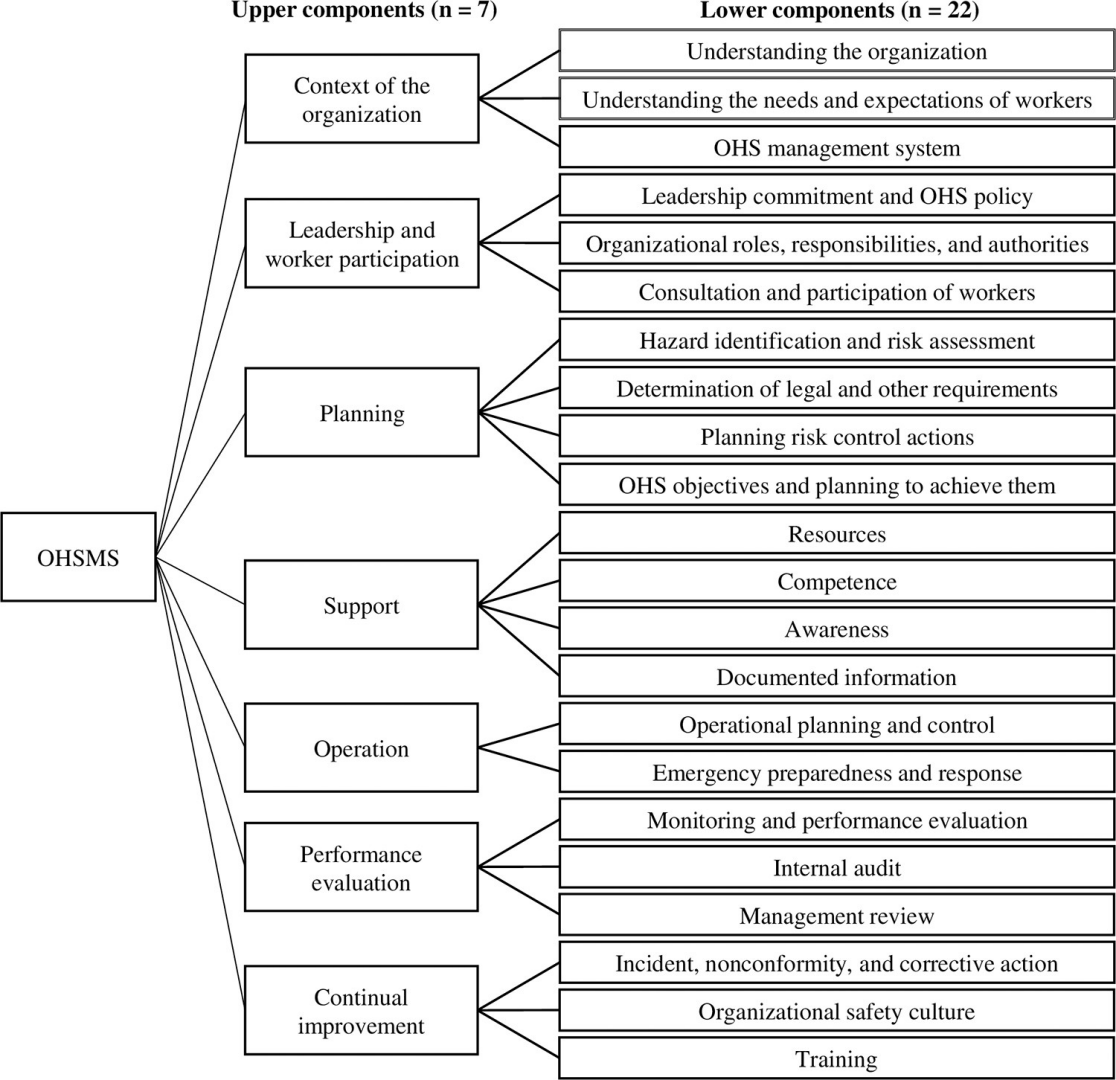
OHSMS standard structuring.

Type B questionnaires were based on KOSHA-MS, a Korean safety management standard developed from international standards, and ISO-45001. Both standards are being used as OHSMS specifications in ROKN workplaces. OHSMS structure was classified into upper (n = 7) and lower (n = 22) components through a preliminary review by 19 naval OHSMS experts and was structured to suit decision-making tools (Fig 2). For the AHP-entropy analysis, the evaluator evaluated the relative importance of the pairwise comparison of the two attributes in the upper and lower components using a 9-point scale (1–9 points, ’two attributes have equal importance’ to ’absolute importance of one attribute’). In addition, for the IPA analysis, the evaluator evaluated the degree to which lower components were performed using a 7-point scale (1–7 points, ’very low’ to ’very high’).

Two types of surveys were conducted online for one month, from February 11 to March 11, 2022. Type A was distributed to workers of the ROKN workplaces, and type B was distributed to naval OHSMS experts. The surveys were approved by the ROKN Headquarters (Culture and Public Relations Division-No.47) and Institutional Review Board of Korea University (KUIRB-2022-0041-01), and informed consent was obtained from all participants before the survey.

The validity and reliability among the variables of each questionnaire were obtained using Kaiser-Mayer-Olkin (KMO), Bartlett’s sphericity test, and Cronbach’s alpha (C_a_). In this study, occupational hazard factors, OHS efforts, and OHSMS components had a KMO value of 0.6 or higher and were all significant in Bartlett’s sphericity test (*P* < 0.001). And all of them showed acceptable confidence levels with C_a_ values of 0.80 or higher.

### Comparison of occupational hazards and OHS efforts by type of workplaces

The ROKN workplaces are divided into four types based on their tasks: repair and maintenance, military engineering, ordnance ammunition, and military logistics. We analyzed the occupational hazard levels and characteristics for each workplace type. OHS efforts were compared and analyzed according to whether OHSMS was applied and the application period. These analyses were conducted within the same work type to minimize the influence of other variables. OHS efforts by OHSMS application were analyzed only for military engineering (OHSMS application rate, 67%) and ordnance ammunition (OHSMS application rate, 25%) types, which include OHSMS applied and unapplied workplaces. As all repair and maintenance workplaces applied OHSMS (OHSMS application rate, 100%), we could not compare the effect depending on whether OHSMS was applied. Therefore, we analyzed OHS efforts by OHSMS operation period in this type. Military engineering and ordnance ammunition workplaces were excluded from the comparative analysis by operation period because they applied OHSMS in similar years, 2019–2020. The military logistics type was excluded from all OHS efforts analyses because no workplaces maintained OHSMS at the time of the study (certification canceled or temporary suspension for standard conversion; OHSMS application rate, 0%). The evaluation scores of occupational hazard factors and OHS efforts were calculated as the sum of scores for each category×100/the sum of the maximum scores that can be used for each category.

### Derivation of OHSMS improvement factors

First, AHP-entropy was used to focus on linearly determining the importance weight and priority of OHSMS components. AHP-entropy is performed in six steps: problem structuring, structural evaluation, AHP analysis, qualitative weight synthesis, entropy-based decisions, and derivation of final weights [26]. As we completed problem structuring according to OHSMS standard specifications, only five steps were conducted from the structural evaluation as follows.

*Step 1*, *structural evaluation*. A pairwise comparison was performed on two factors, *i* and *j* using Saaty’s 9-point scale, and a comparison matrix was created with the derived evaluation value *a*_*ij*_. At this time, *a*_*ij*_ takes the form *a*_*ij*_ = 1/*a*_*ij*_ of the reciprocal number centered on the diagonal of the matrix, and when *i* and *j* are the same, *a*_*ij*_ = 1.

*Step 2*, *AHP analysis*. In this step, the subjective relative importance weight was calculated by solving the matrix eigenvalues of the upper and lower components and checking the consistency of pairwise comparison judgments using the consistency ratio (C.R). Consistency means that the evaluator judged consistently when comparing evaluation elements in pairs. The AHP calculation was performed through the Microsoft Excel Visual Basic for Applications program.

*Step 3*, *weight synthesis*. When the consistency criterion was satisfied (C.R < 0.1), the subjective weights $\left(\omega_{j}^{s}\right)$ of all OHSMS components were calculated by combining the weights of the upper and lower components. In this study, 13 data satisfied the C.R < 0.1.

*Step 4*, *entropy analysis*. In this step, the decision matrix (*x*_*ij*_, *i*, *j* = 1,⋯,m, n; n is the number of evaluation indicators; m is the set of evaluation objects) or entropy analysis is normalized with $p_{ij}=x_{ij}\sum_{i=1}^{m}x_{ij}(i,j=1,\cdots ,m,n)$. Next, the entropy was calculated with $E_{j}=-k\sum_{i=1}^{m}p_{ij}logp_{ij}(k=1/logm,i,j=1,\cdots ,m,n)$ and the objective weight ${(\omega }_{j}^{o})$ was calculated with ${\omega_{j}^{o}=d}_{j}/\sum_{j=1}^{n}d_{j}$ (*j* = 1,⋯, n; as this time, *d*_*j*_ = 1−*E*_*j*_).

*Step 5*, *determination of adjustment weights*. The adjustment weight ($\omega_{j}^{a}$) was derived by combining the subjective weights $\left(\omega_{j}^{s}\right)$ and objective weights ${(\omega }_{j}^{o})$. The highest weight is considered the most important alternative.

Second, IPA was used to evaluate the performance of OHSMS and to derive the improvement factors by identifying low performance compared to importance in the ROKN. The IPA matrix is expressed as quadrants on a two-dimensional plane by setting the *X*-axis to the average of performance evaluated on a 7-point Likert scale and the *Y*-axis to the average of importance derived from AHP-entropy. In the IPA matrix, quadrant 1, ‘concentrate’ is an area that needs intensive improvement due to low performance despite its high importance. Quadrant 2 is a ‘good work’ area with high importance and high performance; quadrant 3 is a ‘low-priority’ area with low importance and low performance; and quadrant 4 is a ‘possible overkill’ area with low importance and high performance.

### Statistical analysis

We tested the normality using the Kolmogorov-Smirnov tests, then employed the Kruskal-Wallis test, post hoc Dunn test, and Mann-Whitney U tests to compare the occupational hazard factors and OHS efforts of the ROKN workplaces. Statistical analysis was performed using SPSS Ver. 26.0, and the significance level for the two-sided test was set at 0.05.

## Results

### Characteristics of study participants

Table 2 shows the characteristics of the 629 ROKN workplaces workers and 29 naval OHSMS experts who participated in the study. Among the ROKN workplaces workers, 49.5% were soldiers (commissioned and enlisted), and 50.5% were civil servants (appointed). The types of their workplaces were repair and maintenance 47.8%, military engineering 21.8%, ordnance ammunition 19.6%, and military logistics 10.8%. In addition, 18% of workers were OHS officers. Among naval OHSMS experts, 68.9% were commissioned, and 31.1% were appointed. Their OHSMS experience period was 1–2 years, the highest at 58.6%. The types of workplaces where they performed OHSMS tasks were repair and maintenance 34.5%, military engineering 44.8%, ordnance ammunition 13.8%, and military logistics 6.9%.

**Table 2:** Characteristics of the study participants.

Category	ROKN workplaces workers	Naval OHSMS experts
Total	629 (100)	29 (100)
Sex		
Male	579 (92.1)	29 (100)
Female	50 (7.9)	0 (0)
Military service duration (years)		
<10	356 (56.6)	9 (31.1)
10–20	108 (17.2)	7 (24.1)
>20	165 (26.2)	13 (44.8)
Military service type		
Commissioned	154 (24.5)	20 (68.9)
Enlistment	157 (25.0)	0 (0)
Appointment	318 (50.5)	9 (31.1)
Workplaces type		
Repair and maintenance	301 (47.8)	10 (34.5)
Military engineering	137 (21.8)	13 (44.8)
Ordnance ammunition	123 (19.6)	4 (13.8)
Military logistics	68 (10.8)	2 (6.9)
OHS officer		
No	516 (82.0)	0 (0)
Yes	113 (18.0)	29 (100)
OHSMS experience period (years)		
<1	179 (28.5)	7 (24.1)
1–2	438 (69.6)	17 (58.6)
>2	12 (1.9)	5 (17.3)

Data were presented as frequencies (percentages).

### Occupational hazard levels and characteristics of the ROKN workplaces

Table 3 shows the occupational hazard levels recognized by workers for each of the four types of workplaces. There was a significant difference in the overall median value of the occupational hazard levels by work type (*P* < 0.001), which was high in the order of repair and maintenance (60.0), military engineering (54.0), ordnance ammunition (47.0), and military logistics (43.5).

**Table 3:** Occupational hazard level in four workplace types.

Occupational hazard levels	Repair and maintenance (n = 301)	Military engineering (n = 137)	Ordnance ammunition (n = 123)	Military logistics (n = 68)	*P*-value
Total	60.0^a^	54.0^b^	47.0^c^	43.5^bc^	< 0.001
	(44.5, 71.0)	(39.9, 62.0)	(31.0, 60.0)	(28.0, 60.0)	
Biochemicals	56.0^a^	48.0^b^	40.0^bc^	36.0^c^	< 0.001
	(36.0, 68.0)	(30.0, 60.0)	(20.0, 60.0)	(20.0, 60.0)	
Physical burden	64.0^a^	60.0^b^	56.0^b^	52.0^b^	< 0.001
	(48.0, 80.0)	(40.0, 68.0)	(40.0, 64.0)	(29.0, 63.0)	
Mechanical and electric	60.0^a^	60.0^a^	46.7^b^	46.7^b^	< 0.001
	(46.7, 73.3)	(40.0, 73.3)	(26.7, 60.0)	(20.0, 66.7)	
Work environment	60.0^a^	51.4^b^	40.0^c^	42.9^bc^	< 0.001
	(45.7, 71.4)	(37.1, 60.0)	(25.9, 60.0)	(25.7, 60.0)	

Data were presented as median (interquartile range). Cases that did not share a common letter (a, b, and c) were significantly different at *P* < 0.001 based on Dunn’s test.

**Fig 3:**
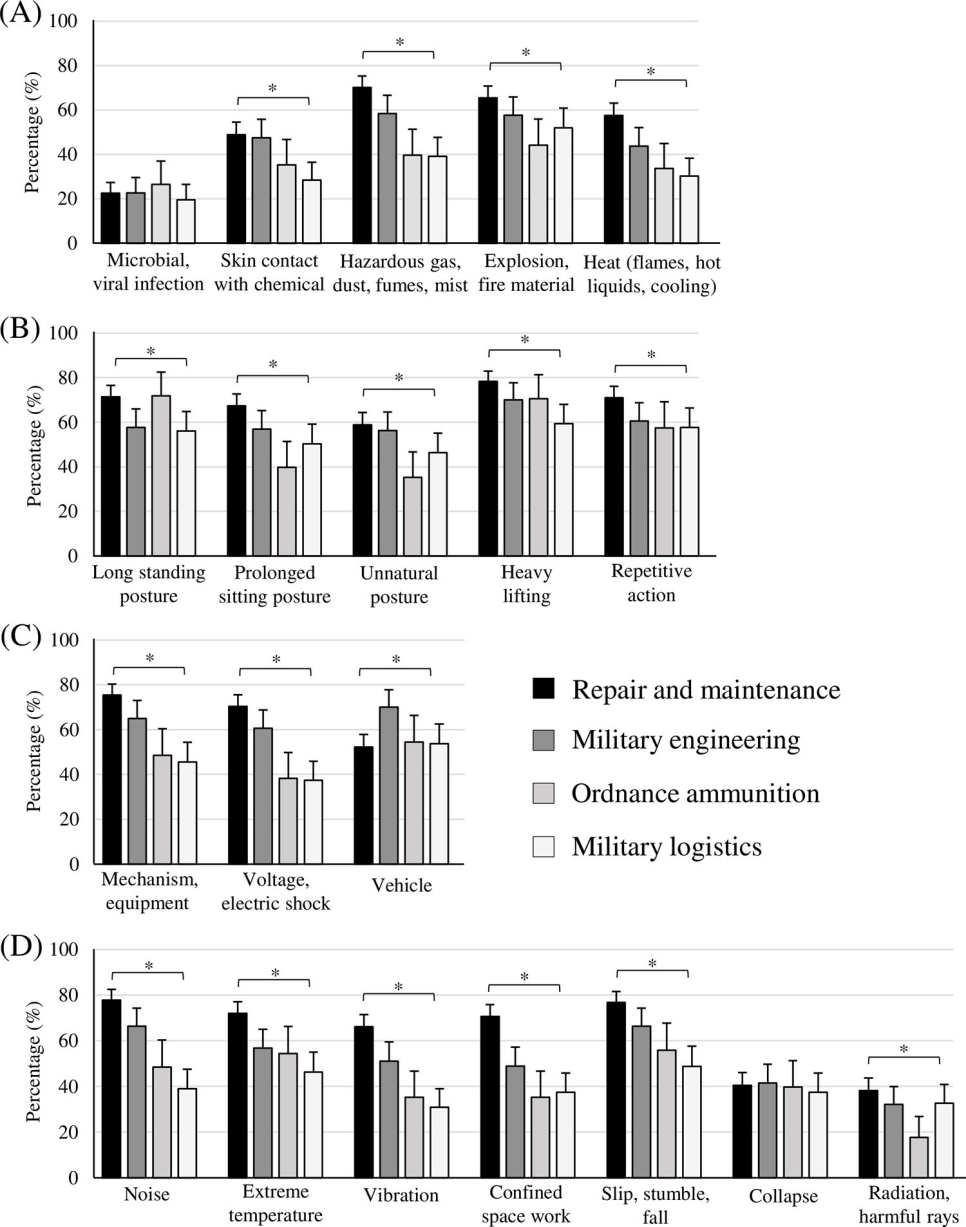
Detailed occupational hazard factors perceived by members of the four different work types in the ROKN workplaces. (A) Biochemicals, (B) Physical burden, (C) Mechanical and electric, and (D) Work environment. Data were presented as the percentage of moderate or high-hazard levels (≥3 points) for each component.

Fig 3. shows the detailed factors of the four occupational hazard areas by work type. All hazard levels except for microbial, viral infection (Fig 3A) and collapse (Fig 3D) showed statistically significant differences between types (*P* < 0.01). In all types, heavy lifting (repair and maintenance, 78.3%; military engineering, 70.0%; ordnance ammunition, 70.5%; military logistics, 59.4%) was the most dangerous (Fig 3B), and the second most dangerous factors were as follows by type: noise (77.8%) in repair and maintenance (Fig 3D), vehicle (70.1%) in military engineering (Fig 3C), long standing posture (71.8%) in ordnance ammunition (Fig 3B), and repetitive action (57.7%) in military logistics (Fig 3B).

### The effectiveness of OHSMS

Table 4 shows the results of comparing OHS efforts between OHSMS applied and unapplied workplaces in military engineering and ordnance ammunition types. The overall median value of OHS efforts was not statistically significantly different between OHSMS-applied workplaces (military engineering, 72.8; ordnance ammunition, 80.0) and unapplied (military engineering, 73.3; ordnance ammunition, 76.7) in either type.

**Table 4:** Comparison of OHS efforts between workplaces to which OHSMS applied and unapplied in military engineering and ordnance ammunition types.

OHS efforts	Military engineering		*P*-value	Ordnance ammunition		*P*-value
	OHSMS	OHSMS		OHSMS	OHSMS	
	applied (n = 110)	unapplied (n = 27)		applied (n = 69)	unapplied (n = 54)	
Total	72.8	73.3	0.900	80.0	76.7	0.168
	(60.0, 83.9)	(60.0, 82.2)		(60.0, 100.0)	(60.0, 80.3)	
OHS system	73.3	70.0	0.762	80.0	76.7	0.109
	(60.0, 83.3)	(60.0, 83.3)		(60.0, 100.0)	(60.0, 80.0)	
OHS management	68.6	68.6	0.900	80.0	72.9	0.178
	(60.0, 82.9)	(60.0, 88.6)		(60.0, 100.0)	(60.0, 80.7)	
Accident prevention activities	80.0	80.0	0.747	80.0	80.0	0.277
	(60.0, 96.0)	(60.0, 88.0)		(60.0, 100.0)	(60.0, 85.0)	

Data were presented as median (interquartile range).

Table 5 shows the results of comparing OHS efforts by operating period (2–5 years) according to the time of OHSMS application (2017–2020) in repair and maintenance types. The overall median value of OHS efforts were 80.0, 80.0, 81.1, and 84.4, respectively, from 2017 to 2020, and there was no statistical difference according to the application period.

**Table 5:** Comparison of OHS efforts by operating period (2–5 years) according to the time of OHSMS application (2017–2020) in repair and maintenance type.

OHS efforts	Year of application				*P*-value
	2017 (n = 43)	2018 (n = 92)	2019 (n = 72)	2020 (n = 94)	
Total	80.0 (73.3, 97.8)	80.0 (68.1, 95.3)	81.1 (67.8, 95.6)	84.4 (76.1, 100.0)	0.165
OHS system	80.0 (76.7, 96.7)	80.0 (67.5, 99.2)	83.3 (66.7, 100.0)	83.3 (70.0, 100.0)	0.548
OHS management	80.0 (65.7, 100.0)	80.0 (65.7, 94.3)	80.0 (65.7, 97.1)	87.1 (71.4, 100.0)	0.106
Accident prevention activities	80.0 (72.0, 100.0)	84.0 (72.0, 96.0)	80.0 (72.0, 100.0)	90.0 (80.0, 100.0)	0.105

Data were presented as median (interquartile range).

### OHSMS importance priority and improvement factors

Table 6 shows the results of the AHP-entropy analysis presenting importance weights and priorities of OHSMS components. The factors with the top 30% (6 factors) importance weight priority were emergency preparedness and response (importance, 0.1465; rank, 1), consultation and participation of workers (importance, 0.0980; rank, 2), resources (importance, 0.0979; rank, 3), leadership commitment and OHS policy (importance, 0.0639; rank, 4), competence (importance, 0.0615; rank, 5), and hazard identification and assessment of risks (importance, 0.0581; rank, 6).

**Table 6:** Adjustment importance weights $\left(\omega_{j}^{a}\right)$ and priorities of OHSMS components through AHP-entropy analysis.

OHSMS components	$\omega_{j}^{s}$ ^a^	$\omega_{j}^{o}$ ^b^	$\omega_{j}^{a}$	Rank
Context of the organization				
Understanding the organization	0.0217	0.0820	0.0421	12
Understanding the needs and expectations of workers	0.0336	0.0549	0.0437	10
OHS management system	0.0155	0.0360	0.0132	22
Leadership and worker participation				
Leadership commitment and OHS policy	0.0581	0.0464	0.0639	4
Organizational roles, responsibilities, and authorities	0.0786	0.0279	0.0520	8
Consultation and participation of workers	0.1163	0.0356	0.0980	2
Planning				
Hazard identification and risk assessment	0.0238	0.1030	0.0581	6
Determination of legal and other requirements	0.0179	0.0370	0.0157	18
Planning risk control action	0.0236	0.0353	0.0197	16
OHS objectives and planning to achieve them	0.0163	0.0366	0.0141	20
Support				
Resources	0.0391	0.1057	0.0979	3
Competence	0.0646	0.0402	0.0615	5
Awareness	0.0337	0.0177	0.0141	21
Documented information	0.0178	0.0350	0.0148	19
Operation				
Operational planning and control	0.1066	0.0176	0.0445	9
Emergency preparedness and response	0.1109	0.0557	0.1465	1
Performance evaluation				
Monitoring and performance evaluation	0.0305	0.0347	0.0250	15
Internal audit	0.0251	0.0440	0.0262	14
Management review	0.0321	0.0252	0.0192	17
Continual improvement				
Incident, nonconformity, and corrective action	0.0312	0.0433	0.0320	13
Organizational safety culture	0.0400	0.0571	0.0541	7
Training	0.0631	0.0291	0.0435	11

^a^$\omega_{j}^{s}$, the AHP-based subjective weight.

^b^$\omega_{j}^{o}$, the entropy-based objective weight.

**Fig 4:**
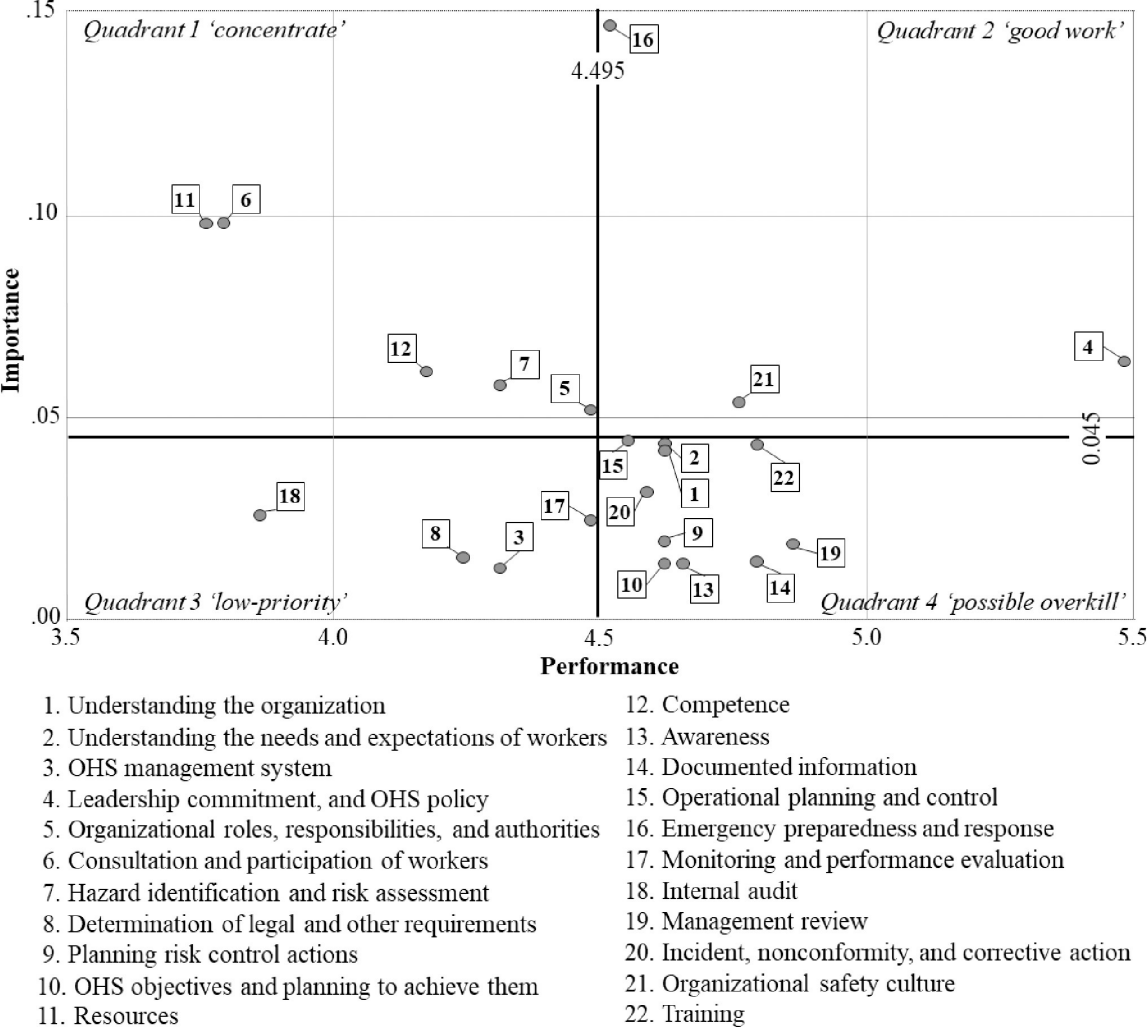
The IPA matrix. The average of X-axis (performance) is 4.495, and the average of Y-axis (importance) is 0.045.

Fig 4 shows the IPA matrix that cross-evaluates the importance and performance of OHSMS attributes. Ten OHSMS components (45.5% of all OHSMS components) were derived from the domain of possible overkill (quadrant 4). Five were derived from the concentrate area (quadrant 1) in the following priorities: consultation and participation of workers (importance, 0.0980; performance, 3.7931), resources (importance, 0.0980; performance, 3.7586), competence (importance, 0.0980; performance, 4.1724), hazard identification and risk assessment (importance, 0.0980; performance, 4.3103), and organizational roles, responsibilities, and authorities (importance, 0.0980; performance, 4.4828). Other attributes were classified as follows: quadrant 2 (good work), three including emergency preparedness and response, and quadrant 3 (low-priority), four including internal audit.

## Discussion

This study identified the impact of OHSMS on safety performance in the ROKN workplaces and the improvement factors for improving OHSMS effectiveness. OHSMS-applied workplaces did not show a statistically higher level of OHS efforts than those unapplied. We also identified no better OHS efforts in workplaces with longer OHSMS application periods. When we evaluated improvement factors for OHSMS in the ROKN, five were derived in the order of; consultation and participation of workers; resources; competence; hazard identification and risk assessment; and organizational roles, responsibilities, and authorities.

This study conclusively confirmed that OHSMS did not improve OHS efforts in the ROKN workplaces (Tables 4 and 5). Although the effectiveness of OHSMS has been empirically proven, some organizations still show insufficient effectiveness [14, 27]. Heras-Saizarbitoria et al. (2019) and Ghahramani et al. (2019) said that simple OHSMS certification does not guarantee safety performance, and efforts are needed to identify hazard factors at the organization’s site and reflect them in OHSMS activities [14, 27]. In OHSMS-certified companies that lack such efforts, series accidents (Hyundai Development Company collapse accident; Samlip&Shany, Paris Croissant, Companies casualty damage, etc.), continue to occur until recently, revealing problems with the implementation of OHSMS. Especially in the ROKN, it is more difficult to identify and act on nonconformities in OHSMS because most industrial sectors are closely linked to military elements, and external assessors cannot evaluate all work processes and risks. In this study, the ROKN had different hazard levels and characteristics at each workplace type and showed various industrial environments (Table 3, Fig 3). If these hazards of each workplace have not been properly considered, OHSMS may simply be a standard form favorable for certification. Furthermore, as the ROK government legally regulates the establishment of a safety management system from 2021, OHSMS certification and acquisition of safety titles have become important. This social background has influenced the adoption of OHSMS by many Korean companies [1], and it is possible that ROKN also applied OHSMS to form a safe image and improve external reliability. When OHSMS is used to meet social needs and responsibilities, there may not be a correlation between OHSMS effectiveness and safety performance [14], as shown in the results of this study. Additionally, this issue would have made ROKN focus on increasing the number of OHSMS-certified workplaces rather than reflecting on whether OHSMS was applied appropriately for site risks. In conclusion, ROKN should address the lack of effectiveness caused by improper implementation of OHSMS and improve it as a practical safety tool.

This study presented five factors for the improvement of OHSMS in ROKN (Fig 4). The highest priority among them was consultation and participation of workers (Table 6). Fernández-Muñiz et al. (2012a, 2012b), in their study of a wide range of companies in Spain, described this factor as key to successfully securing a company’s OHS capabilities [28, 29]. They said that since the goal of OHSMS is to eliminate or reduce risk, sharing risk information perceived by workers who are in direct contact with risk factors in the workplace is of paramount importance to achieving OHSMS [28, 29]. This is even more important given the diverse industrial environment of the ROKN that we identified earlier (Table 3, Fig 3). The ROKN must evaluate each workplace’s hazards in more detail to identify those that are vulnerable to employee health and safety and determine how much should be improved [30]. Therefore, the ROKN will be able to improve safety capabilities more effectively if it improves the workers’ participation in connection with the fourth improvement factor, hazard identification and risk assessment. However, OHSMS tends to be recognized as the unique task of the person in charge, so this participation is not yet well performed [31]. Besides, the ROKN has a more vertical and closed organizational atmosphere than companies, so workers might not actively communicate their risks to each other. Therefore, the ROKN should establish detailed strategies to stimulate worker participation in OHSMS activities. Examples include developing and evaluating indicators for measuring safety culture and climate [30, 32, 33], providing incentives for safety and risk propositions [34], or citing proven ’worker involvement incentive programs’ [35].

Meanwhile, resources, competence, organizational roles, responsibilities, and authorities were other improvement factors in quadrant 1 (Fig 4). Unlike the results of this study, these factors were relatively well-performed in general companies [21, 22]. This difference can be explained for the following reasons. First, unlike hiring OHS experts in companies, the ROKN determines OHSMS personnel through work assignments. As a result, non-experts often perform OHSMS tasks. In addition, due to the nature of military personnel, their positions are frequently changed every 1 to 2 years, making it challenging to gain stable experience and maintain expertise. Even in this study, the average working period of OHSMS experts was only 1.56 years (Table 2). Therefore, in the ROKN, human and technical support will be needed to establish competency standards for OHSMS performers and to ensure conditions for implementing OHSMS, such as strengthening professional education and hiring skilled personnel. For this support to be effective, the reorganization of OHSMS should also be considered. Systemic improvement is needed, such as giving tasks to members with relatively low job shift rates so they can be skilled in OHSMS tasks for a long time or applying different job shift duration to OHSMS officers.

In a situation where the importance of national defense safety from industrial accidents is being emphasized, this study is meaningful in that it diagnosed OHSMS effect of the ROKN for the first time and drew improvement measures. This study suggested results contributing to the industrial safety of the ROKN, which will be important information for the ROKN to monitor and evaluate OHSMS activities in the future to develop the system in the right direction. On the other hand, despite these advantages, this study has several limitations. First, because of the characteristic of the military, the respondent may have evaluated the occupational hazard levels low and OHS efforts high, fearing that their evaluation would cause adverse results or disadvantages to the organization. Therefore, the actual conditions of application of OHSMS in the ROKN may be slightly different from the results of this study. Second, only subjective safety performance indicators were used, because of the limitation of available data. Cooper and Phillips (2004) suggested using both objective indicators (time lost, accident rate, etc.) and subjective indicators (safety mood, safety effort, etc.) to evaluate the safety effect [36], and a study [27] using both indicators yielded different results in each case. However, to overcome these limitations, we have secured the consistency and reliability of the results by analyzing the safety performance from various angles depending on OHSMS’s application status and the operating period. In the future, follow-up studies using more diverse safety performance indicators should be conducted based on this study.

## Conclusion

More and more ROKN workplaces are adopting OHSMS, but little has been confirmed about the empirical effectiveness of this standard system. This study confirmed that OHSMS did not lead to safety performance in the ROKN and derived five OHSMS attributes they should focus on to improve this problem. These results can be used as primary data for ROKN to implement OHSMS effectively and adequately to prevent industrial accidents. In addition, these results may encourage more armed forces to check the OHSMS and could provide a usable evaluation model. Therefore, we look forward to further studies targeting more advanced and broader OHSMS-certified units than this study.

## Supporting information

S1 File The characteristics of study participants and entire ROKN workplace workers. (DOCX)

## Abbreviations

AHP: Analytic Hierarchy Process
C_a_: Cronbach’s Alpha
C.R: Consistency Ratio
IPA: Importance-Performance Analysis
KMO: Kaiser-Mayer-Olkin
KOSHA: Korea Occupational Safety and Health Association
OHS: Occupational Health and Safety
OHSMS: Occupational Health and Safety Management System
ROK: Republic of Korea
ROKN: Republic of Korea Navy
