# Supplementary material for: Evaluation of effectiveness and improvement factors of occupational health and safety management system in the Republic of Korea Navy based on AHP-entropy and IPA
Source: PLoS One. 2023 Apr 4;18(4):e0283653. doi: 10.1371/journal.pone.0283653 (PMC10072467; doi:10.1371/journal.pone.0283653)
Supplement: S1 File — (DOCX) [file pone.0283653.s001.docx]

**Supporting Information**

**Evaluation of Effectiveness and Improvement Factors of Occupational Health and Safety Management System in the Republic of Korea Navy based on AHP-entropy and IPA**

Sang ji Lee^1,2^, Yun-Hee Choi^2,3^, Da-An Huh^4^, Seok Joon Yoon^4^, Kyong Whan Moon^2,3,5 *^

^1^ Republic of Korea Navy Headquarters, Republic of Korea

^2^ Department of Health and Safety Convergence Science, Korea University, Anam-ro 145, Seongbuk-gu, Seoul, 02841, Republic of Korea

^3^ BK21 FOUR R&E Center for Learning Health System, Korea University, Anam-ro 145, Seongbuk-gu, Seoul, 02841, Republic of Korea

^4^ Institute of Health Sciences, Korea University, Anam-ro 145, Seongbuk-gu, Seoul, 02841, Republic of Korea

^5^ Department of Health and Environmental Science, Korea University Anam-ro 145, Seongbuk-gu, Seoul, 02841, Republic of Korea

* Corresponding author

E-mail: kwmoon@korea.ac.kr

**S1 Table. The characteristics of study participants and entire ROKN workplace workers.**

| Category | ROKN workplace workers in this study | | Entire ROKN workplace workers | *p*-value^a^ |
| --- | --- | --- | --- | --- |
|  | N | Percentage (%) | Percentage (%) |  |
| Sex |  |  |  |  |
| Male | 579 | 92.1 | 93.0 | 0.937 |
| Female | 50 | 7.9 | 7.0 |  |
| Military service duration (years) |  |  |  |  |
| <10 | 356 | 56.6 | 75.4 | 0.074 |
| 10–20 | 108 | 17.2 | 11.3 |  |
| >20 | 165 | 26.2 | 13.3 |  |
| Military service type |  |  |  |  |
| Commissioned | 154 | 24.5 | 27.6 | 0.238 |
| Enlistment | 157 | 25.0 | 22.5 |  |
| Appointment | 318 | 50.5 | 49.9 |  |
| Workplaces type |  |  |  |  |
| Repair and maintenance | 301 | 47.8 | 50.0 | 0.415 |
| Military engineering | 137 | 21.8 | 20.0 |  |
| Ordnance ammunition | 123 | 19.6 | 20.0 |  |
| Military logistics | 68 | 10.8 | 10.0 |  |

^a^Fisher’s exact test.
